# “Vivir el paso a paso”: Itinerarios de rehabilitación de la niñez con discapacidad en narrativas de mujeres cuidadoras en la zona poniente de Santiago de Chile

**DOI:** 10.18294/sc.2025.5792

**Published:** 2025-11-27

**Authors:** Nelson Muñoz-Lizana, Paulina Osorio-Parraguez, Juan Andrés Pino-Morán

**Affiliations:** 1 Autor de correspondencia. Doctor en Salud Pública. Fonoaudiólogo, Departamento de Fonoaudiología, Facultad de Medicina, Universidad de Chile. Investigador joven, Núcleo Milenio Estudios en Discapacidad y Ciudadanía (DISCA), Santiago, Chile. nelsonmunoz@uchile.cl Universidad de Chile Departamento de Fonoaudiología Facultad de Medicina Universidad de Chile Santiago Chile nelsonmunoz@uchile.cl; 2 Doctora en Sociología. Profesora titular, Departamento de Antropología, Facultad de Ciencias Sociales, Universidad de Chile, Santiago, Chile. posorio@uchile.cl Universidad de Chile Departamento de Antropología Facultad de Ciencias Sociales Universidad de Chile Santiago Chile posorio@uchile.cl; 3 Doctor en Sociología. Profesor asociado, Instituto de Ciencias de la Salud, Universidad de O’Higgins, Rancagua, Chile. Investigador principal, Núcleo Milenio Estudios en Discapacidad y Ciudadanía (DISCA), Chile. juan.pino@uoh.cl Universidad de O’Higgins Instituto de Ciencias de la Salud Universidad de O’Higgins Rancagua Chile

**Keywords:** Ruta Terapéutica, Niños con discapacidad, Cuidadores, Servicios de rehabilitación, Salud Colectiva, Chile

## Abstract

En Chile, la rehabilitación infantil se reconoce como un derecho respaldado por marcos normativos. Sin embargo, su implementación presenta limitaciones que restringen el acceso y la continuidad de los procesos, y las experiencias y estrategias de las mujeres cuidadoras usuarias del sistema han recibido escasa atención en el diseño y evaluación de políticas públicas. En este contexto, este artículo caracteriza los itinerarios terapéuticos de rehabilitación de niñeces con discapacidad en la zona poniente de Santiago de Chile, desde la perspectiva de sus cuidadoras principales. Entre agosto de 2023 y marzo de 2024, a partir de una metodología cualitativa con enfoque etnográfico, se realizaron acompañamientos con observación participante, registrados en notas de campo, y entrevistas semiestructuradas a 13 mujeres. Los resultados evidencian trayectorias fragmentadas, atravesadas por múltiples barreras de acceso, alta carga de gestión familiar y discontinuidades en la atención. Se identifican cuatro momentos clave: sospecha y diagnóstico, tránsito institucional, desarrollo de intervenciones, y alta terapéutica. Las cuidadoras sostienen los procesos de atención mediante estrategias cotidianas que articulan apoyos públicos, comunitarios y privados. En este marco, el artículo discute los límites de las políticas públicas actuales y resignifica la rehabilitación como una práctica contextualizada, sostenida en redes de cuidado afectivas y desiguales.

## Introducción

La rehabilitación infantil se define como un proceso planificado, intersectorial y sostenido en el tiempo, orientado a optimizar el funcionamiento, el desarrollo y el bienestar de niñas y niños con condiciones de salud que presentan o se encuentran en riesgo de experimentar discapacidad^1^^,^^2^. En consonancia con la Clasificación Internacional del Funcionamiento, la Discapacidad y la Salud para la Infancia y Adolescencia (CIF-IA)^3^, la Organización Mundial de la Salud reconoce la rehabilitación como un componente esencial de las estrategias orientadas a garantizar el derecho a la salud de la niñez con discapacidad. Desde un enfoque de equidad e inclusión, estas estrategias buscan reducir barreras, promover entornos accesibles y fomentar la participación activa^4^^,^^5^. Para ello, se requiere contar con marcos normativos sólidos, financiamiento adecuado y servicios integrales, disponibles y pertinentes para las comunidades, que aseguren un acceso equitativo y de calidad^5^. 

No obstante, en América Latina y el Caribe, la implementación efectiva de la rehabilitación infantil sigue siendo limitada, debido a persistentes brechas estructurales, desigualdades sociales y fragmentación institucional^6^^,^^7^. Investigaciones realizadas en distintos países de la región, principalmente en Brasil, han documentado numerosas barreras que afectan el acceso y la calidad de estos servicios: diagnósticos errados y/o tardíos^8^^,^^9^^,^^10^^,^^11^, listas de espera prolongadas^12^^,^^13^, discontinuidades en la atención^8^, débil coordinación intersectorial^8^^,^^13^^,^^14^, escaso reconocimiento del rol de las cuidadoras y desatención de sus necesidades específicas^8^^,^^9^, escasez de recursos humanos y limitaciones materiales^13^, entre otras. Con frecuencia, la carga de sostener los procesos de rehabilitación recae de manera desproporcionada en las familias, especialmente en las mujeres, quienes deben emprender una búsqueda activa, y muchas veces solitaria, por servicios fragmentados, emocionalmente exigentes y desarticulados entre sí. En este trayecto, deben articular recursos públicos, privados y comunitarios, al tiempo que sostienen prácticas de cuidado que desbordan las prescripciones médicas, en un escenario marcado por desigualdades de clase, género, etnia y territorio^13^.

En Chile, este panorama adopta características particulares que reflejan muchas de las tendencias descritas. Según el III Estudio Nacional de la Discapacidad (ENDISC III)^15^, un 14,7% de niños, niñas y adolescentes presenta alguna discapacidad, con una mayor prevalencia en sectores de bajos ingresos. No obstante, solo el 14,9% de esta población accede a servicios de rehabilitación, a pesar de contar con un marco legal que lo garantiza (Ley 20422)^16^, lo que evidencia una brecha significativa entre el derecho formal y las condiciones efectivas para ejercerlo. Aunque existen programas como el Fondo de Intervenciones de Apoyo al Desarrollo Infantil (FIADI), implementado en el marco del subsistema Chile Crece Más y algunas iniciativas locales, gran parte del proceso de rehabilitación continúa centralizado en recintos hospitalarios especializados o delegado a instituciones privadas con financiamiento público, entre ellas, la Fundación Teletón, caso paradigmático del modelo asistencialista en el país y en la región^17^^,^^18^. Esta configuración expone las limitaciones estructurales del sistema que comprometen la continuidad del cuidado y profundizan las desigualdades en salud en contextos de trayectorias de atención prolongadas. Además, las tra­yectorias de las familias usuarias de los servicios de re­habilitación han sido escasamente consideradas para el diseño y evaluación de políticas públicas sobre la mate­ria, particularmente aquellas trayectorias que consideran saberes, estrategias y tensiones que enfrentan las cuidadoras en su vínculo con el sistema de salud. Indagar en estas experiencias resulta clave para vislumbrar las dinámicas que generan las barreras estructurales y para orientar políticas que garanticen el acceso y la continuidad de la rehabilitación desde la perspectiva de las propias familias.

Comprender cómo se configuran estos recorridos requiere un enfoque que exceda la descripción institucional. En este sentido, la noción de itinerarios terapéuticos resulta especialmente útil para analizar cómo se producen estas trayectorias en contextos de desigualdad y fragmentación como en Chile. Lejos de constituir rutas lineales, los itinerarios se conciben como recorridos complejos, atravesados por interrupciones, afectos, aprendizajes y decisiones^19^^,^^20^. Desde esta perspectiva, el abordaje de los procesos salud-enfermedad-atención (PSEA)^21^, donde se incluye la rehabilitación^22^, se desarrolla en escenarios en los que coexisten diversos modelos de atención que se entrelazan en prácticas concretas de cuidado^23^. Por ello, resulta clave indagar en el conjunto de espacios en que se desarrolla la vida de la niñez con discapacidad y su entorno para comprender cómo se produce la rehabilitación y qué significados adquiere.

Tal como han señalado estudios recientes en salud colectiva, como los de Venturiello^24^^,^^25^^,^^26^ y Brage^27^, este análisis implica atender no solo a los dispositivos formales disponibles, sino también a las estrategias de navegación institucional, las resistencias cotidianas, los aprendizajes afectivos y el papel central que asumen las cuidadoras (madres, abuelas u otras mujeres del entorno familiar) como agentes que median entre saberes, gestionan recursos, interpretan indicaciones clínicas y sostienen vínculos médico-terapéuticos en condiciones de precariedad institucional. A partir de estas consideraciones, el presente estudio se propone caracterizar los itinerarios de rehabilitación de la niñez con discapacidad en la zona poniente de Santiago de Chile, a partir de las narrativas de sus cuidadoras principales. Se pone énfasis en los sentidos atribuidos al proceso terapéutico, las estrategias desplegadas para sostener el cuidado más allá de los dispositivos formales, y las tensiones emergentes en la relación con actores institucionales. Al visibilizar estas experiencias, se busca aportar a una comprensión crítica de la rehabilitación infantil en condiciones de desigualdad estructural, y contribuir al diseño de políticas públicas que reconozcan el cuidado como una práctica colectiva y vital para la garantía del derecho a la salud.

### Itinerarios terapéuticos de la rehabilitación de la niñez con discapacidad: aportes desde la salud colectiva y los estudios críticos de la discapacidad

Este estudio se inscribe en una perspectiva que articula aportes de la salud colectiva y de los estudios críticos de la discapacidad para pensar los procesos de atención en salud como fenómenos complejos, atravesados por relaciones de poder, condiciones materiales, marcos institucionales y saberes diversos. La noción de PSEA^21^ constituye un punto de partida para comprender la rehabilitación no como un conjunto aislado de intervenciones biomédicas, sino como parte de un entramado de prácticas, sentidos y relaciones sobre la salud y la discapacidad infantil que se configuran en el universo sociocultural donde transcurre la vida cotidiana. En estos escenarios, coexisten y se interrelacionan múltiples modelos terapéuticos que, desde la perspectiva del pluralismo médico, dan forma a estrategias singulares de cuidado^23^^,^^28^. 

En este marco, la noción de itinerario terapéutico constituye una herramienta conceptual clave para analizar los procesos de atención en salud desde la perspectiva de quienes los transitan. De acuerdo con Alves y Souza^29^, desde un enfoque comprensivo, el itinerario terapéutico se concibe como una construcción relacional y contextual, compuesta por el conjunto de decisiones, estrategias, significados y prácticas que las personas movilizan en su búsqueda de cuidado frente a una necesidad de salud. Se trata de un proceso dialógico e intersubjetivo, en el que confluyen distintos actores sociales, saberes, vínculos afectivos y modelos de atención, dentro de marcos sociohistóricos específicos que condicionan y posibilitan las trayectorias del PSEA^20^^,^^30^. En contextos donde se anticipa una oferta de atención fragmentada y desigual, la categoría de itinerario terapéutico permite explorar cómo las cuidadoras de la niñez con discapacidad, con los recursos disponibles y redes de apoyo, construyen y negocian sentidos, traducen indicaciones clínicas y articulan saberes familiares, comunitarios y terapéuticos para sostener el proceso de rehabilitación. 

Desde una perspectiva relacional, se reconoce que los procesos de cuidado a la niñez con discapacidad no pueden disociarse de las condiciones materiales, simbólicas y afectivas que atraviesan la vida de quienes cuidan^31^^,^^32^. Lejos de ser un rol naturalizado, el cuidado se configura como un campo de disputas políticas y epistémicas, en el que se tensionan marcos de reconocimiento, distribución de recursos, legitimidad de saberes y visibilidad social del trabajo de cuidado. En América Latina, diversos estudios han mostrado cómo las mujeres (madres, abuelas y otras figuras familiares) no solo acompañan los procesos terapéuticos, sino que actúan como mediadoras activas entre los dispositivos institucionales y las necesidades de las personas cuidadas^10^^,^^26^^,^^33^. Esta mirada permite comprender el cuidado como una práctica interdependiente, inserta en redes sociales y familiares que coorganizan la vida cotidiana en torno a la discapacidad y sostenida en condiciones estructuralmente desiguales^13^.

Estas discusiones adquieren especial relevancia cuando se aborda la rehabilitación infantil como un campo atravesado por discursos normalizadores y exigencias de adherencia funcional. Desde los estudios críticos de la discapacidad y las infancias^34^^,^^35^^,^^36^^,^^37^, se ha señalado cómo los modelos rehabilitadores tradicionales tienden a organizar las vidas de las niñeces con discapacidad en torno a trayectorias prescritas de desarrollo, demandando la corrección de desvíos respecto de un ideal de funcionalidad. Estas expectativas no solo operan sobre los cuerpos de la niñez, sino también sobre las familias, que muchas veces deben adaptarse a marcos institucionales rígidos que transforman la vida doméstica en un espacio de intervención profesional constante. En este sentido, se problematiza el lugar que ocupa la familia -y en particular las madres- en la cadena de responsabilidades terapéuticas, así como los efectos que estas demandas producen en sus trayectorias de vida^33^^,^^38^^,^^39^^,^^40^^,^^41^.

Perspectivas latinoamericanas plantean que la discapacidad no constituye una condición exclusivamente individual, sino una experiencia relacional que se configura en el entramado de interacciones entre personas, instituciones y territorios^34^. Así, pensar la rehabilitación desde los itinerarios terapéuticos implica reconocer que este proceso se inscribe en un campo de disputas -entre saberes, modelos y actores- donde lo terapéutico se redefine más allá de la consulta profesional. Como han mostrado algunos estudios en la región^11^^,^^13^^,^^24^^,^^25^^,^^26^^,^^27^, estas trayectorias están profundamente atravesadas por relaciones de género, clase, territorio y afectividad, configurando una pedagogía práctica del cuidado que es, al mismo tiempo, una forma de resistencia y una vía de producción de saberes desde la experiencia vivida en contextos de desigualdad estructural.

## Aspectos metodológicos

Este estudio adopta un enfoque cualitativo, orientado a comprender los itinerarios terapéuticos de rehabilitación infantil desde la perspectiva de quienes sostienen cotidianamente los procesos de cuidado. Se inscribe en el paradigma interpretativo^42^, que reconoce estas trayectorias como construcciones intersubjetivas, situadas en contextos atravesados por relaciones de poder, significados culturales y estructuras institucionales. Bajo un enfoque etnográfico^43^, se realizaron entrevistas semiestructuradas, observaciones participantes y notas de campo, lo que permitió captar tanto los relatos como las interacciones, silencios y prácticas de las cuidadoras. La muestra se construyó mediante muestreo teórico-intencional y técnica bola de nieve, incluyendo 13 cuidadoras principales (12 madres y una abuela), a cargo de niños y niñas entre 1 y 9 años con discapacidad permanente, residentes en la zona poniente de Santiago y usuarias del sistema público de salud.

El trabajo de campo se desarrolló entre agosto de 2023 y marzo de 2024, e incluyó visitas domiciliarias, acompañamientos a centros de rehabilitación y registros situados en contextos comunitarios. Se realizaron 30 entrevistas, de entre 60 y 90 minutos, grabadas y transcritas íntegramente. El guion fue flexible, orientado a la reconstrucción narrativa en torno al acceso, vínculos institucionales y sentidos de los procesos terapéuticos. Se llevaron a cabo principalmente en los hogares o espacios cotidianos para las participantes, lo que permitió anclar los relatos en los escenarios donde se producen los cuidados y las dinámicas familiares. Estos contextos favorecieron un clima de confianza y construcción de narrativas detalladas, aunque en algunos casos implicaron limitaciones asociadas a la variabilidad en las condiciones y tiempos de realización o a la presencia de otros miembros del grupo familiar, aspectos considerados en el análisis e interpretación de los datos. El análisis fue temático, con codificación abierta, axial y selectiva mediante ATLAS.ti, en diálogo con principios de la teoría fundamentada^44^. Se elaboraron diagramas gráficos y narrativas de los itinerarios, que se validaron con las participantes, fortaleciendo la coherencia interpretativa.

La reflexividad incluyó el reconocimiento del investigador como fonoaudiólogo inserto en el sistema público y como hombre en un campo feminizado. Esta posición requirió un ejercicio crítico sostenido, que incluyó discusiones colectivas, análisis de notas de campo y validación de hallazgos.

El estudio contó con aprobación del Comité de Ética de Investigación en Seres Humanos de la Facultad de Medicina de la Universidad de Chile (Proyecto N.º 027-2023), y todas las participantes fueron informadas previamente sobre los objetivos, alcances y procedimientos del estudio, incluidas las condiciones de grabación, transcripción y uso académico de los relatos. La confidencialidad se resguardó mediante la asignación de códigos alfanuméricos (por ejemplo: C1), sin vínculo con datos personales, y mediante la eliminación de referencias que pudieran permitir la identificación de las participantes o de las niñeces. Los registros fueron almacenados en dispositivos protegidos y de acceso restringido, manteniéndose la anonimización durante todo el proceso de codificación y presentación de resultados. Estas medidas aseguraron la privacidad de las participantes y la coherencia analítica del material utilizado.

Los resultados que se informan son parte del trabajo doctoral del autor principal para optar al grado de Doctor en Salud Pública de la Universidad de Chile, cuya investigación mayor se titula “Procesos de rehabilitación de niñeces con discapacidad de la zona poniente de Santiago de Chile: una aproximación a las experiencias de sus cuidadoras principales desde los itinerarios terapéuticos”.

## Resultados

A partir del análisis de las narrativas de las cuidadoras principales, se reconstruyen cuatro momentos clave de los itinerarios terapéuticos de rehabilitación de la niñez con discapacidad: 1) la sospecha y confirmación de que algo ocurre: las experiencias que dan inicio al itinerario; 2) la peregrinación para llegar a rehabilitación: transiciones institucionales, tiempos de espera y autogestión; 3) el desarrollo de las intervenciones terapéuticas: sentidos, tensiones y resignificación de la rehabilitación; y 4) la continuidad y proyecciones abiertas del proceso rehabilitador: la tensión entre la intermitencia de los apoyos y el alta terapéutica. Estos momentos no deben entenderse como etapas cronológicas rígidas, sino como categorías analíticas que permiten caracterizar las complejidades de los itinerarios del proceso de rehabilitación, considerando su diversidad y las particularidades de cada caso.

### La sospecha y confirmación de que algo ocurre: las experiencias que dan inicio al itinerario

Los itinerarios de rehabilitación analizados en este estudio suelen comenzar durante los primeros años de vida. En algunos casos, se activan a partir de diagnósticos en etapas pre o perinatales, frecuentemente asociados a condiciones genéticas o prematurez. En otros, la sospecha emerge en la vida cotidiana, a través de una percepción reiterada por parte de las cuidadoras o del entorno cercano de que “algo no anda bien”. Si bien difieren en su origen, ambas experiencias marcan un punto de inflexión en la trayectoria familiar, dando inicio a un proceso de búsqueda, incertidumbre y reorganización de la vida cotidiana.

Los diagnósticos tempranos, lejos de vivirse como un acto meramente clínico, se configuran como lo que Osorio Carranza denomina disrupciones biográficas^45^: momentos que alteran profundamente las expectativas, las temporalidades y los roles familiares, junto con redefinir el estatus de la niñez con discapacidad para el sistema de salud y su familia. A su vez, el conocimiento del diagnóstico para las cuidadoras implica una doble o triple preocupación, sobre todo en los momentos de hospitalización prolongada posterior al parto, pues deben gestionar los cuidados de otros integrantes de la familia menores de edad y evaluar los recursos disponibles para los insumos médicos en contextos de desempleo o trabajos informales.

En este marco, las cuidadoras relatan experiencias marcadas por tensiones con equipos médicos, muchas veces expresadas en clave de violencia obstétrica^46^, desconfianza o propuestas que desestiman sus convicciones y emociones. La figura médica aparece como portadora de un saber tecnocrático que, en ocasiones, impone decisiones sin considerar las situaciones que están viviendo las cuidadoras.

“*Me dijeron que la ley me amparaba y que podía abortar, porque mi guagua no iba a nacer viva. Con ese síndrome* [trisomía 18] *los niños no vivían. Yo les dije que no, que si la guagua se iba a morir, yo lo aceptaba, pero no estaba dispuesta a matarla*”. (C5)

“*La matrona me dijo que mi embarazo era de muy alto riesgo, que era casi imposible que llegara a término, y que ni siquiera me iba a hacer carnet. Le pedí una orden para una ecografía, y me dijo: ‘Sí, pero no te hagas expectativas, capaz que ni siquiera esté’*”. (C6)

“*Me preguntaron si la quería o no, así tal cual: ‘*¿Va a querer a la niña o la va a dar en adopción?*’. Les dije que estaban locas, que era mi hija y que no la iba a dejar*”. (C8)

En este punto, se observa la coexistencia de procesos de atención y desatención^23^^,^^47^, donde las indiferencias o descréditos por parte de algunos profesionales se pueden considerar como manifestaciones de una desatención estructural que reproduce jerarquías de clase, género y saber^47^. Frente a estas situaciones, las mujeres despliegan estrategias de resistencia, como rechazar recomendaciones médicas, buscar segundas opiniones o activar redes familiares para costear exámenes particulares, cuando los recursos económicos y el tipo de previsión de salud se lo permite. Estas acciones no solo expresan agencia, sino que posibilitan sostener decisiones afectivas y éticas en condiciones de precariedad. 

En otros casos, el itinerario no parte de un diagnóstico médico, sino de una sospecha sostenida que emerge en la vida doméstica. Estas señales suelen manifestarse entre los 6 y 24 meses, cuando las cuidadoras, a través de la observación, la comparación o la intuición, perciben que el desarrollo de sus hijos no responde a las expectativas normativas.

“*Todo normal, tomaba su leche, fue un niño para mí sano hasta cuando ya tenía como cinco o seis meses, que yo lo dejaba ahí en la cama y ahí se quedaba. Si lo ponías de lado, se iba para los lados, y ahí encontramos que no afirmaba su cabecita*”. (C1)

Estas señales no siempre son recibidas con certeza. A menudo, se transitan entre la negación, la duda, el autoengaño o la presión externa. Las redes familiares pueden sostener emocionalmente a las cuidadoras, pero también imponer mandatos que las hacen responsables del diagnóstico o del “*no haberlo visto antes*”. Como han analizado ​​Runswick-Cole *et al*.^39^^,^^40^, sobre ellas recae un imperativo de vigilancia y gestión del desarrollo infantil que define su valoración social como madres.

“*Yo no le hacía caso porque era guagua… Al año y cinco meses, las tías de parte de mi pareja me decían: ‘Eso no es normal’. Pero yo decía: es normal, porque me dijeron que venía sano*”. (C4)

El sistema de salud, en lugar de acoger estas sospechas, muchas veces las deslegitima. Al igual que lo reportado por Puga *et al*.^11^, las experiencias de minimización institucional retrasan diagnósticos oportunos y limitan el acceso a intervenciones tempranas. Esto no solo amplifica la incertidumbre, sino que profundiza los procesos de desatención planteados por Hersh-Martínez^47^.

“*La doctora del* [nombre del hospital] *me dijo que era pura mañana, que era un niño completamente sano*”. (C3)

“*En el hospital tuve mala suerte las primeras veces. La primera vez la vieron así no más: ‘Son cosas de abuelas’, dijeron. La segunda neuróloga dijo: ‘No le voy a hacer nada para que no llore’, y no le hicieron nada*”. (C2)

Frente a la falta de diagnósticos certeros, demoras de atención, el trato despersonalizado y las negociaciones entre el saber experto y el saber experiencial de las cuidadoras, estas últimas toman un rol activo en la búsqueda de prestaciones de salud que se adapten a sus requerimientos. Consultan por fuera del sistema público, interpretan informes, buscan orientación en redes informales y registran el comportamiento de sus hijos para acumular evidencia. Estas acciones, descritas por Brage como tácticas del cotidiano^27^, reflejan una forma de sostener el cuidado desde la experiencia, incluso sin un diagnóstico oficial, y en medio de la fragmentación institucional.

“*Quise pedir una segunda opinión con la neuróloga. Cada vez que me tocaba control con ella, me daba un diagnóstico diferente: pasaban cuatro o cinco meses y cambiaba de opinión… Fuimos al neurólogo particular porque queríamos una derivación para ver si era posible que ingresara a la Teletón. Yo no quería que perdiera lo que ya había aprendido. Quería que siguieran ayudándola o al menos que me dijeran a mí cómo seguir trabajando con ella*”. (C13)

De este modo, en lugar de limitarse a resistir, las cuidadoras transforman la desatención en aprendizaje práctico y afectivo que contribuye a desarrollar lo que Pava-Ripoll denomina capital emocional^33^, entendido como una forma de saber afectivo que combina reflexión, intuición y acción para afrontar las problemáticas del proceso. 

Así, esta primera etapa del itinerario no solo inaugura la relación con el sistema de atención, sino que constituye una experiencia formativa y constitutiva del rol cuidador. Emociones ambivalentes como angustia, culpa o alivio se entrelazan con acciones prácticas de contención, búsqueda y defensa. Como sugiere Goodley^36^, en estos contextos el diagnóstico, o su ausencia, se convierte en un hito que no puede reducirse a una marca de déficit y expresión de vulnerabilidad, pues implica una serie de reconfiguraciones afectivas, redefiniciones de prioridades familiares, disputas de sentido y esfuerzos que revelan un potencial transformador para la vida cotidiana y modifican el lugar de la discapacidad dentro de ella.

### La peregrinación para llegar a rehabilitación: transiciones institucionales, tiempos de espera y autogestión

Tras la sospecha inicial o el diagnóstico, las familias ingresan a una etapa marcada por un intenso recorrido fragmentado a través de distintos niveles del sistema de salud y educación, en búsqueda de apoyos terapéuticos adecuados. El acceso a la rehabilitación se ve condicionado por factores estructurales, como la previsión de salud, la articulación territorial de los servicios, la disponibilidad de redes personales y la capacidad de decodificar la información en un sistema opaco. Algunas cuidadoras logran ingresar a través de derivaciones desde el primer nivel de atención o servicios hospitalarios, mientras otras dependen de referencias informales, profesionales conocidos o atenciones particulares. 

Esta etapa del itinerario puede entenderse como una “peregrinación institucional”, en la que las cuidadoras asumen tareas que exceden el rol pasivo de usuarias, ya que coordinan interconsultas, gestionan documentos y traslados, y ofrecen contención emocional al grupo familiar. En línea con lo planteado por Alves^19^ y Menéndez^23^, estos modos de navegación no constituyen simples desplazamientos entre servicios, sino espacios donde distintas racionalidades, médica, doméstica y burocrática, se intersectan y dan lugar a formas concretas de cuidado. De este modo, el peregrinaje no solo evidencia las ineficiencias del sistema, sino que muestra cómo las familias coproducen la atención y participan activamente en la producción social del proceso de rehabilitación en contextos de fragmentación.

Un hallazgo central en esta etapa es la función del diagnóstico médico como llave de acceso a terapias y servicios sociales. Más que una herramienta clínica, el diagnóstico se convierte en una credencial institucional que legitima la demanda y determina el tipo de apoyos disponibles. Este fenómeno se inscribe en los procesos de legitimación o validación de la demanda descritos por Gerhardt^20^, Venturiello^24^ y Brage^27^, en los que el reconocimiento de una necesidad de cuidado depende más de la capacidad de cumplir con los requisitos burocráticos que de la urgencia o la complejidad del caso. De este modo, las familias se ven forzadas a acumular legitimidad ante el sistema para ser reconocidas como portadoras de una demanda válida, lo que redefine el sentido mismo del proceso de atención.

“*Me dijo: ‘Lo que pasa es que no podemos ingresarlo sin un diagnóstico, un informe, un examen o algo similar’. Entonces yo le respondí que estaba bien, y ella me dijo: ‘Si puede volver a llevarlo a un neurólogo* [privado], *sería bueno. Si no, igual lo pondremos en lista de espera para el médico, pero eso puede tardar mucho tiempo. Si tiene la posibilidad de llevarlo por fuera, hágalo’*”. (C11)

Por su parte, el sistema público de salud, debilitado por la escasez de recursos y las extensas listas de espera, fomenta de manera implícita una articulación con prestadores privados, incluidos aquellos del sector privado no lucrativo (fundaciones y corporaciones que operan con financiamiento estatal), como estrategia de compensación institucional. Como consecuencia, se intensifica la carga económica y logística sobre las familias, que deben endeudarse o activar redes personales para acceder a una atención oportuna, lo que profundiza las desigualdades sociales y territoriales.

“*Cuando terminó la atención en la fundación, pensé: ‘¿Y ahora qué?’. Había que esperar que nos contactaran nuevamente para saber qué hacer con mi hijo, a qué terapeuta llevarlo. Luego fuimos con la neuróloga y me dijo: ‘Esto ya no me corresponde, ahora es algo genético’. Pero no hay nada disponible en lo genético, entonces hay que hacerlo particular*”. (C1)

El gasto de bolsillo se instala como umbral de acceso. Las familias enfrentan pagos por exámenes, traslados o sesiones particulares. Esta privatización silenciosa del cuidado ocurre en un contexto de precariedad, donde las políticas públicas no garantizan continuidad efectiva. Esta modalidad de funcionamiento, frecuentemente naturalizada como un complemento necesario, refuerza lo que Menéndez^23^ y Hersch-Martínez^47^ denominan desatención estructural, entendida como una forma de privatización encubierta del cuidado que traslada a los hogares la responsabilidad de garantizar la continuidad terapéutica.

“*El examen de ADOS* [*Autism Diagnostic Observation Schedule* o Escala de Observación para el Diagnóstico del Autismo] *costaba entre 140, 130, 120 mil pesos. Y ella, creo que me cobró 100 mil. Si no lo hacía, no podía llevarlo a la terapeuta*” [$100.000 CLP equivalen a $110 USD según tipo de cambio promedio 2023-2024]. (C7)

“*Me dijo que tendría que hacerlo por mi cuenta. Entonces fui a un instituto que me dijeron que era el lugar más especializado. El primer examen costó 200 mil pesos, el otro 400 mil. El último costó como 900 mil, y salió normal*”. (C1)

Ante esta desprotección, las cuidadoras despliegan diversas estrategias: se inscriben en múltiples centros, presentan reclamos, recurren a contactos, buscan atajos o reorganizan sus finanzas. Estas acciones expresan una forma de agencia construida en la desatención que, como plantea Venturiello^26^, configura una pedagogía del cuidado, es decir, un saber práctico y relacional que se aprende en la experiencia y permite sostener el acompañamiento más allá de las prescripciones institucionales. Esta capacidad de actuar en la incertidumbre también involucra una dimensión afectiva y ética que, en diálogo con Pava-Ripoll^33^, forma parte y contribuye al capital emocional de las cuidadoras, al movilizar energía y compromiso para mantener el cuidado frente a la falta de apoyo estructural. No obstante, estos aprendizajes implican costos físicos y morales que revelan los límites de esa agencia.

“*Yo soy la que pide las horas, la que explica todo cuando cambian de terapeuta, la que pelea por las interconsultas. A veces siento que soy más la asistente social que la mamá*”. (C6)

“*Ya estaba muy agotada, frustrada. Incluso llamé al Ministerio de Salud para averiguar si podían hacer algo, porque de verdad estaba colapsada. Averiguamos cuánto salían las terapias particulares y era algo así como 40 mil pesos por sesión, tres veces a la semana. Era carísimo, pero yo pensaba: ‘No importa, vemos cómo lo hacemos darle las terapias que necesita. Haremos lo que tengamos que hacer para pagar eso’*”. (C11)

Este tránsito también deja huellas emocionales y físicas. Las cuidadoras narran síntomas de agotamiento, insomnio, ansiedad, dolor corporal. La culpa aparece como una constante: por no haber actuado antes, por no poder con todo, por sentir que siempre falta algo. Estas experiencias confirman que el cuidado se produce en un terreno de tensiones. Si bien Thomas^48^ lo concibe como una práctica ética orientada a sostener la vida y la interdependencia, las narrativas aquí recogidas evidencian los límites de esa ética cuando el cuidado se ejerce sin las condiciones materiales ni institucionales para sostenerlo. En este sentido, Kittay^31^ amplía el debate al situar la dependencia y el apoyo al cuidador como dimensiones centrales de la justicia relacional, al sostener que no hay cuidado posible si quienes cuidan no son, a su vez, cuidados. Las voces de las participantes revelan, por tanto, una paradoja relevante en la que el mismo acto que preserva la vida también produce desgaste, mostrando que la ética del cuidado requiere ser acompañada por políticas que reconozcan y sostengan su dimensión material y colectiva.

“*Pasaron semanas que yo no dormí en ningún momento. Entonces la neuróloga me dijo que descansara*”. (C6)

“*Estoy cansada, pero si no lo hago yo, nadie lo hace*”. (Nota de campo, acompañamiento a C2).

Esta etapa no se reduce a un trámite previo. Se trata de una experiencia compleja, donde el desgaste institucional se cruza con aprendizajes forzados, decisiones éticas y redes de sostenimiento. En este recorrido, las cuidadoras no solo enfrentan barreras materiales, sino que reformulan sus vínculos con el sistema de salud y reconfiguran las formas de habitar el cuidado, atravesadas por mandatos sociales, desigualdades acumuladas y disponibilidad afectiva que, al mismo tiempo que sostiene la rehabilitación, expone sus límites.

### El desarrollo de las intervenciones terapéuticas: sentidos, tensiones y resignificación de la rehabilitación

Tras la primera etapa de movilización institucional, las cuidadoras ingresan a un nuevo terreno: el de las intervenciones terapéuticas. Lejos de representar un momento de contención, este tramo se experimenta como una extensión de las exigencias previas. Las instituciones participantes incluyen una variedad de centros, tanto públicos como privados: Centros de Salud Familiar (CESFAM), programas municipales, hospitales de referencia, e institutos nacionales, fundaciones privadas, profesionales que ejercen de manera independiente, entre otros. En algunos casos, los tratamientos se realizan en el hogar, a través de modalidades domiciliarias. Las sesiones, lejos de estar garantizadas, dependen de múltiples factores: disponibilidad de profesionales, criterios diagnósticos, convenios de financiamiento y, sobre todo, la gestión cotidiana de las familias. A esto se suman los tiempos acotados de atención y la frecuente rotación de profesionales, lo que debilita la continuidad terapéutica.

“*Antes no pasaba eso. La kinesióloga anterior me daba todas las horas del mes y atendía de forma continua, durante casi un año. En cambio, la nueva profesional nos incluyó en un plan de solo ocho sesiones. Si no hay avances, te dejan fuera*”. (C5)

A estas limitaciones se suman otras barreras que obstaculizan el ejercicio efectivo del derecho a la rehabilitación. Las dificultades para acceder a las sesiones incluyen distancias excesivas entre el domicilio y los centros, falta de transporte accesible, imposibilidad de reorganizar horarios por trabajos informales, ausencia de acompañantes, o colisión con otras actividades escolares o médicas programadas.

“…*y así a veces perdimos la hora* [de atención], *porque a veces me tocan trayectos de tres horas para llegar al instituto. En el metro lo más complicado son los ascensores que muchas veces están en mal estado y tengo que llegar hasta la otra estación*” (notas de campo, acompañamiento a C9).

“*Él sigue necesitando más apoyo, y una sesión con la fonoaudióloga cada dos semanas no es suficiente. Me han dicho que, si puedo buscar más ayuda, lo haga. Pero hasta ahora no tengo ni el tiempo ni el dinero para pagar más terapias, ni para que lo vengan a ver a la casa. Si yo lo llevo, pierdo la tarde completa de trabajo. Además, dejo de trabajar y tengo que encargarme también de mi otra hija*”. (C4)

Estas dificultades se ven agravadas por la necesidad de cofinanciar tratamientos. Las terapias particulares, los traslados, la alimentación durante los recorridos y los materiales para ejercicios en casa representan costos de bolsillo significativos. Pese a que las familias deberían contar con atención garantizada por el sistema público, en la práctica deben asumir gastos permanentes para asegurar continuidad y calidad del proceso.

“*En ese tiempo no recibíamos pensión, así que buscamos que FONASA ofreciera más cobertura, no en dinero, sino en cantidad de sesiones y horas. Compramos muchas sesiones de kinesiología y, como íbamos a un centro médico, el papá compraba bonos a su nombre para que atendieran a mi hija. También, comprábamos bonos a nombre de la hermana, yo misma, mi mamá… para que no perdiera sus terapias*”. (C6)

“*Entre pasajes, comida y las sesiones, se me va la plata de la semana, así que organizo algunas cosas para juntar dinero. Entre mi marido y yo hacemos rifas, vendemos completos, cualquier cosa para tener ingresos*”. (Nota de campo, acompañamiento C5).

Las experiencias de atención en rehabilitación infantil se ven afectadas por programas con cobertura limitada según edad o recursos disponibles, lo que genera rotaciones frecuentes de profesionales y cambios en los enfoques terapéuticos. En este escenario, las cuidadoras elaboran valoraciones diversas: en algunos casos, destacan la construcción de vínculos basados en la empatía, la escucha activa y el reconocimiento de su rol, lo que permite generar espacios de confianza y contención emocional en medio de la inestabilidad. En otros, enfrentan relaciones tensas con los equipos técnicos, marcadas por rigidez, trato impersonal o desvalorización de su experiencia, lo que refuerza una sensación de exclusión y discontinuidad en el proceso de atención. Como advierten Venturiello^25^^,^^26^ y Gerhardt^20^, el itinerario terapéutico no se sostiene únicamente en prescripciones técnicas, sino en negociaciones cotidianas en las que se disputa el reconocimiento del rol cuidador. Frente a estas tensiones, las cuidadoras despliegan estrategias para hacer valer su voz: observan, comparan, adaptan indicaciones y, cuando lo consideran necesario, interrumpen o modifican el tratamiento.

“*Yo le decía a la terapeuta que ella ya había pasado por esa etapa, pero la trataban como si recién empezara. Ella se sentía aburrida, hacía los ejercicios rápido solo para terminar. A veces es difícil que los profesionales escuchen, como si uno no supiera lo que ha vivido con ellos*”. (C2)

La rehabilitación no se restringe a lo prescrito por los servicios: se vive y se ajusta en función de lo cotidiano, de lo que se percibe como útil, posible o sostenible. En este contexto, las familias la expanden hacia la casa, la escuela o la comunidad, integrando actividades cotidianas, juegos, estimulación sensorial o ejercicios adaptados. También recurren a terapias complementarias como la hipoterapia o el uso regular de piscinas, en su mayoría autogestionadas o facilitadas por redes locales. Estas prácticas, en diálogo con el pluralismo médico^21^^,^^28^, muestran cómo las familias combinan saberes biomédicos, familiares y comunitarios para configurar nuevas formas de atención. Así, las cuidadoras articulan experiencias y conocimientos y convierten la rehabilitación en una práctica distribuida orientada a sostener el cuidado dentro de los márgenes de lo posible.

“*Cuando tenía como un año y dos meses, comenzamos con la hipoterapia. Es como con la terapeuta: pasamos por procesos donde nos dan el alta, luego de medio semestre nos vuelven a llamar, y así seguimos. A ella le encanta y le sirve harto*”. (C6)

“*Una amiga me invitó a un taller en una piscina temperada, donde los sábados prestan tres pistas para niños con discapacidad... Desde el primer día, fue feliz en el agua. Se soltaba solo, flotaba con los ‘tallarines’, y todos se reían. Le hizo muy bien, sobre todo para la musculatura. Las sesiones eran de dos horas*”. (C1)

En este entramado de esfuerzos, los establecimientos educacionales también se configuran como espacios donde se sostiene, parcialmente, la dimensión terapéutica. Algunas familias encuentran en la escuela, a través del apoyo de profesionales de la salud o docentes diferenciales, una continuidad que muchas veces no logran obtener en los centros de salud. No obstante, aunque estos dispositivos son valorados por las cuidadoras, su funcionamiento no está diseñado ni cuenta con las condiciones necesarias para reemplazar los procesos de rehabilitación, lo que tensiona las expectativas depositadas en la escuela y refleja la fragilidad del entramado institucional de los servicios.

“*Hasta ahora solo tiene fonoaudióloga en el colegio. No me ha mandado el cuaderno, así que ahora va a tener que empezar a mandar el cuaderno con la estimulación*”. (C1)

“*En el colegio, la tía* [terapeuta] *me dijo que ha avanzado mucho. Cuando recién entró, ya se sabía las vocales. Un día ella las puso en la pizarra y él las dijo todas: a-e-i-o-u. Me emocioné, porque estuve meses reforzándolas en la casa. Para mí, eso es un gran logro*”. (C3)

El desarrollo de las intervenciones terapéuticas se configura como un proceso extendido y no lineal que desborda los márgenes institucionales. Así, para estas familias, rehabilitar no significa solo asistir a sesiones formales, sino articular diversas acciones que se distribuyen entre la casa, la escuela, la comunidad y los centros de salud. En este entramado, las familias combinan apoyos, estrategias y recursos, integrando lo cotidiano en lo terapéutico y adaptándolo a las condiciones de posibilidad y sostenibilidad en contextos marcados por la desigualdad. Este movimiento transforma el sentido mismo de la discapacidad, pues ya no se orienta únicamente a corregir o compensar un déficit, sino a reorganizar la vida en torno a otras formas de valor, relación y agencia. En esta línea, Goodley^36^ y Runswick-Cole^39^ sostienen que la discapacidad puede entenderse como un espacio de producción social de afectos y vínculos, donde la dependencia se reinterpreta como interdependencia y se amplían las formas posibles de autonomía. Desde esta perspectiva, las prácticas de las cuidadoras revelan una potencia afirmativa, ya que mientras sostienen la rehabilitación también reinventan modos de vida y reconstruyen la noción de desarrollo fuera del ideal normativo.

### La continuidad y las proyecciones abiertas del proceso rehabilitador: la tensión entre la intermitencia de los apoyos y el alta terapéutica

La mayoría de las trayectorias de este estudio abarcan procesos rehabilitadores prolongados, muchas veces superiores a cuatro años. Las cuidadoras relatan avances, retrocesos y detenciones, en un tránsito que no sigue un patrón lineal. El proceso se interrumpe y retoma, cambia de lugar o se adapta, según el desarrollo de cada persona, las decisiones clínicas y las propias percepciones familiares. Durante las entrevistas, se identificaron distintas formas de cierre o suspensión. Algunas responden al alta formal, cuando se considera superada una etapa o resuelta la necesidad de atención. Otras aparecen como interrupciones temporales, vinculadas a la rotación de profesionales, la falta de cupos o la saturación institucional. En varios casos, las cuidadoras interpretan el alta no como una evaluación integral, sino como una decisión administrativa. También se describen interrupciones voluntarias por parte de las familias, motivadas por la percepción de escasos avances o desacuerdo con el enfoque terapéutico.

Frente a esta situación, las cuidadoras despliegan estrategias de continuidad que desafían el cierre formal del proceso. Buscan nuevas derivaciones, pagan sesiones privadas, contactan terapeutas conocidos o fortalecen apoyos desde el entorno escolar y comunitario. Lo terapéutico, lejos de terminar, se desplaza, cambia de escenario, se transforma en modalidad, se inscribe en otros vínculos.

“*Ahora que la tía lo dio de alta estoy buscando una fundación que sea gratuita, porque no puedo costear ese tipo de gastos semanalmente. Es mucho dinero, considerando que son varias terapias. Por ejemplo, si necesita fonoaudióloga, terapeuta ocupacional, son aproximadamente 50 mil pesos a la semana, pero yo no tengo los recursos*”. (C7)

Esta persistencia puede leerse, en el sentido planteado por Menéndez^23^, como una forma de autoatención ampliada, entendido como un proceso colectivo que reconfigura los límites del sistema y permite sostener el cuidado más allá de su cobertura formal. En estas dinámicas, el trabajo cotidiano de las cuidadoras mantiene la continuidad que las instituciones interrumpen, configurando una red paralela, en la que la rehabilitación se coproduce dentro y fuera del sistema.

En esta fase, las expectativas sobre el futuro y el sentido del proceso adquieren un lugar central. Con el tiempo, la meta se desplaza desde la normalización hacia acompañamientos situados y metas funcionales alcanzables, donde cada avance cotidiano tiene valor propio. Esta transformación implica también una nueva forma de comprender la discapacidad, que deja de centrarse en el déficit y se orienta hacia los ritmos particulares de cada niñez. Esta transformación implica una comprensión relacional y afectiva de la discapacidad, cercana a la noción de *dis/ability* de Goodley^37^, en la que la diferencia se asume como parte de la vida y no como carencia a superar. Sin embargo, este cambio no es absoluto ni uniforme. La aceptación convive con ambivalencias, frustraciones o dificultades para asumir que ciertas condiciones no desaparecerán.

“*Con estos niños es todo paso a paso. No se trata de hacerse grandes ilusiones, sino de acompañarlos en lo que ellos van logrando. Como mamás queremos lo mejor, pero es mejor agradecer cada avance, por pequeño que sea, que frustrarse esperando algo más. En mi caso, veo a mi hijo y para mí está sano: no necesita medicamentos, come bien, está activo. Es eso: vivir sus logros con él y buscar apoyo donde se pueda*”. (C1)

“*Hasta el día de hoy me cuesta. Me cuesta mucho aceptar que ella no está enferma, que esto es una condición, que lo va a seguir haciendo. Eso es lo que más me cuesta*”. (C2)

De este modo, aunque los dispositivos clínicos establezcan un punto de cierre, los itinerarios terapéuticos para acompañar el desarrollo de la niñez con discapacidad siguen su curso más allá de las instituciones. Las familias, con los recursos disponibles, median entre el ideal clínico del alta y la continuidad del cuidado. En términos de Osorio Carranza^45^, la rehabilitación se integra a la trayectoria biográfica familiar y se prolonga como un proceso vital más que como un episodio sanitario, adaptándose a lo posible y a lo significativo de cada experiencia. 

## Discusión

Los itinerarios terapéuticos de rehabilitación infantil en la zona poniente de Santiago, tal como emergen en las narrativas de las cuidadoras, muestran que la rehabilitación no puede entenderse como un recorrido lineal ni exclusivamente clínico. Se configura más bien como una práctica social, política y afectivamente densa, atravesada por interrupciones, decisiones morales y disputas de sentido. En diálogo con los aportes latinoamericanos sobre itinerarios terapéuticos, los hallazgos confirman que las familias no se limitan a transitar entre servicios, sino que coproducen la rehabilitación mediante estrategias afectivas, éticas y organizativas que compensan la desatención institucional^19^^,^^20^^,^^21^^,^^23^^,^^24^^,^^27^. En esta perspectiva, la rehabilitación se comprende como un proceso relacional que combina saberes profesionales y familiares, sostenido por aprendizajes, vínculos y recursos que las familias movilizan en contextos desiguales.

En el caso chileno, aunque el país adhiere a los marcos internacionales de derechos de las personas con discapacidad y establece un marco normativo sobre rehabilitación^16^, el ejercicio de ese derecho sigue condicionado por exigencias burocráticas, dependencia del diagnóstico, tipo de previsión, localización territorial y capacidad económica de las familias. Ello produce circuitos de acceso que dependen de la capacidad familiar para gestionar derivaciones, activar redes y financiar prestaciones, y no de garantías universales, lo que reproduce la desatención estructural descrita por Menéndez^23^ y Hersch-Martínez^47^. Este patrón coincide con hallazgos en otros contextos latinoamericanos, donde se evidencian demoras diagnósticas, discontinuidades y débil articulación intersectorial^9^^,^^10^^,^^11^. Sin embargo, mientras en países como Brasil el Sistema Único de Salud ha logrado cierta articulación entre niveles de atención, en Chile la lógica subsidiaria del sistema produce una “tercerización de lo público”, delegando la continuidad terapéutica a fundaciones y prestadores privados. A diferencia de los escenarios descritos por Pedrosa *et al*.^8^, en los que las redes públicas intentan articular programas comunitarios, la experiencia chilena se desarrolla en un sistema mixto y fragmentado, donde la rehabilitación depende en gran medida del esfuerzo doméstico y la capacidad de pago familiar. Ello configura una forma de privatización encubierta del cuidado que refuerza desigualdades de clase y género, sin los contrapesos institucionales observados en otros países de la región^10^^,^^27^.

En este escenario, las cuidadoras asumen un papel decisivo en la sostenibilidad del proceso. Durante la búsqueda de atención, sus itinerarios se asemejan a un peregrinaje institucional en el que coordinan derivaciones, gestionan documentos y cubren costos de tratamiento. Este trabajo, invisibilizado por las políticas sanitarias, constituye una forma de agencia política que transforma la desatención en acción. En diálogo con el estudio de Venturiello sobre itinerarios terapéuticos^24^, las mujeres elaboran lo que puede entenderse como pedagogías del cuidado, saberes prácticos y relacionales aprendidos en la experiencia, que permiten sostener los procesos más allá de las capacidades estatales. 

El análisis de las intervenciones terapéuticas muestra que las cuidadoras amplían la noción de rehabilitación al incorporar prácticas domésticas, comunitarias y escolares, como juegos, ejercicios, terapias alternativas y apoyos educativos, que configuran un proceso distribuido y plural, más allá de la prescripción biomédica^25^. Este hallazgo se inscribe en el pluralismo médico planteado por Menéndez^21^ y retomado por Venturiello^25^, en el que las racionalidades biomédicas, familiares y comunitarias coexisten y se reconfiguran según las condiciones locales. En este contexto, el ámbito comunitario, aunque menos visible que el institucional, se consolida como espacio donde se sostienen procesos de atención, contención y acompañamiento. Talleres extraprogramáticos, redes informales, centros vecinales y prácticas de cooperación entre mujeres configuran entramados paralelos de cuidado. 

No obstante, a diferencia de experiencias brasileñas que han integrado terapias comunitarias en programas públicos^8^, en el contexto chileno, las redes familiares y comunitarias existentes presentan un escaso reconocimiento institucional y la continuidad se construye bajo incertidumbre. Las familias no están aisladas, pero su acción colectiva carece de validación pública. Más que apoyos secundarios, estas iniciativas expresan una dimensión colectiva del cuidado que sostienen los procesos en los márgenes del sistema formal. Estas diferencias muestran los diversos matices de expresiones latinoamericanas del cuidado en contexto de discapacidad infantil, en la que se entretejen cuidados relacionales y colectivos, con experiencias más individualizadas y privatizadas. En ambas, la feminización del trabajo de sostén y las desigualdades estructurales se mantienen como ejes comunes que condicionan el acceso, la continuidad y el sentido del proceso terapéutico. Desde la salud colectiva, reconocer su potencial implica integrarlas como actores legítimos en la producción social de salud.

En coherencia con esta lectura, Meyer *et al*.^1^ y Negrini *et al*.^2^ proponen entender la rehabilitación como un proceso intersectorial, sostenido y orientado al bienestar, más que como una suma de intervenciones clínicas. Aunque los marcos internacionales y nacionales reconocen la rehabilitación como derecho y estrategia integral, las trayectorias chilenas revelan una institucionalidad que no articula salud, educación y cuidado social, sino que fragmenta responsabilidades y limita la coordinación. Desde esa perspectiva, se hace visible la brecha entre el ideal normativo y su implementación práctica, que expone la distancia entre el derecho formal y su ejercicio efectivo en contextos de desigualdad. Sin embargo, las trayectorias chilenas revelan una institucionalidad que fragmenta responsabilidades y carece de coordinación entre salud, educación y cuidado social. Este desajuste entre la definición normativa y su aplicación práctica muestra la distancia entre el derecho formal y su ejercicio efectivo en contextos de desigualdad. 

Hacia el cierre del itinerario, muchas cuidadoras desplazan sus expectativas de recuperación hacia una comprensión de la rehabilitación como acompañamiento vital, más allá de la normalización^34^^,^^49^. Este cambio dialoga con los planteamientos de Osorio Carranza^45^ sobre las trayectorias biográficas del cuidado y con los enfoques que conciben la discapacidad como una experiencia relacional y afectiva^36^. Desde esta perspectiva, las prácticas de las familias desafían la noción de desarrollo “normal” que estructura buena parte de las intervenciones terapéuticas y educativas. Como advierten Remorini y Rowensztein^49^, la idea de normalidad en el desarrollo infantil no es un hecho biológico, sino una construcción histórica que orienta modos de clasificación, medición y jerarquización de los cuerpos y los aprendizajes. Los hallazgos de este estudio muestran cómo las cuidadoras resisten a ese marco normativo, al valorar los avances cotidianos según los ritmos y posibilidades de cada niño o niña, en lugar de los parámetros clínicos de progreso. En este sentido, la resignificación de la rehabilitación en las trayectorias chilenas no implica solo una adaptación a la escasez de recursos, sino una redefinición ética y epistemológica del cuidado que cuestiona las jerarquías de saber y las expectativas de normalización.

Aunque el presente estudio no tuvo como propósito realizar un análisis comparativo sistemático por perfiles de atención, se observaron diferencias relevantes en los itinerarios según el tipo de programa, el acceso a redes familiares ampliadas o la modalidad educativa en que participaban las niñeces (escuelas especiales, proyectos de integración escolar u otros). Las cuidadoras con mayores vínculos comunitarios o inserción en espacios organizados de discapacidad (como fundaciones o centros vecinales) mostraban una mayor capacidad de gestión, circulación y contención emocional, lo que confirma la relevancia de las redes sociales como determinantes del itinerario^26^. Estas experiencias refuerzan la necesidad de fortalecer los espacios comunitarios de rehabilitación en las políticas públicas como componentes legítimos de las redes de salud, reconociendo su papel en la producción colectiva del bienestar. Este punto constituye una línea analítica a profundizar en futuros estudios, en particular, considerando cómo estas variables median las posibilidades reales de continuidad del proceso terapéutico.

En términos de política pública, estos hallazgos cuestionan el alcance real de programas como Chile Crece Más o las redes locales de rehabilitación, cuya implementación se ve obstaculizada debido a la falta de coordinación intersectorial y al peso de los criterios administrativos. El diagnóstico se transforma en una llave de acceso más que en parte del acompañamiento, y la cobertura sigue determinada por el tramo de previsión y la capacidad de pago de las familias. Esta situación, también observada por Puga *et al*.^11^ en otros países de la región, adquiere en Chile una intensidad particular por la debilidad del sistema público y la concentración de la oferta en instituciones privadas con financiamiento estatal. La atención primaria, con limitada capacidad resolutiva en discapacidad infantil, no logra articular el cuidado continuo, mientras los servicios especializados se concentran en centros de alta complejidad con cobertura restringida y criterios de alta dispares. Desde la salud colectiva, esta constatación evidencia que la universalidad formal del derecho a la rehabilitación no asegura justicia sanitaria si no se reconocen las dimensiones relacionales y comunitarias del cuidado en contextos de desigualdad estructural. 

El carácter cualitativo del estudio y su foco territorial limitan la posibilidad de generalización, aunque permiten comprender dimensiones afectivas, morales y políticas difíciles de captar desde otros enfoques. Las observaciones acompañadas aportaron profundidad a la comprensión de los itinerarios, pero sería necesario ampliar su alcance para capturar las interacciones institucionales en tiempo real. Asimismo, futuros estudios podrían comparar trayectorias entre distintos territorios o modalidades de atención, para explorar cómo los factores socioeconómicos y de red influyen en la continuidad del cuidado.

A modo de conclusión, los itinerarios analizados evidencian que la continuidad de la rehabilitación no depende del sistema de salud, sino del trabajo cotidiano, afectivo y ético de las cuidadoras. En contextos de fragmentación institucional y desigualdad, las familias coproducen la rehabilitación mediante estrategias de gestión, contención y aprendizaje que sostienen los procesos donde el Estado se ausenta. Esta experiencia revela que la rehabilitación no es una práctica técnica, sino una acción social y política que redefine los significados de discapacidad, cuidado y ciudadanía. Frente a ello, se hace necesario avanzar hacia políticas públicas que reconozcan el valor del cuidado como dimensión constitutiva del derecho a la salud y fortalezcan las redes comunitarias como espacios legítimos de producción de bienestar. Garantizar la rehabilitación implica también garantizar las condiciones sociales que hacen posible cuidar.
