# Supplementary material for: “Vivir el paso a paso”: Itinerarios de rehabilitación de la niñez con discapacidad en narrativas de mujeres cuidadoras en la zona poniente de Santiago de Chile
Source: Salud Colect. 2025 Nov 27;21:e5792. [Article in Spanish] doi: 10.18294/sc.2025.5792 (PMC12788990; doi:10.18294/sc.2025.5792)
Supplement: Translation

# Translation: “Living step by step”: Rehabilitation pathways of children with disabilities in the narratives of women caregivers in western Santiago, Chile

**Authors:** Nelson Muñoz-Lizana, Paulina Osorio-Parraguez, Juan Andrés Pino-Morán

**Affiliations:** 1 Department of Speech, Language and Hearing Sciences, Faculty of Medicine, Universidad de Chile. Young researcher, Núcleo Milenio Estudios en Discapacidad y Ciudadanía (DISCA), Santiago, Chile. nelsonmunoz@uchile.cl; 2 Doctor of Sociology. Full Professor, Department of Anthropology, Facultad de Ciencias Sociales, Universidad de Chile, Santiago, Chile. posorio@uchile.cl; 3 Doctor of Sociology. Associate Professor, Institute of Health Sciences, O'Higgins University, Rancagua, Chile. Principal Investigator, Núcleo Milenio Estudios en Discapacidad y Ciudadanía (DISCA), Chile. juan.pino@uoh.cl

**Keywords:** Therapeutic Itinerary, Children with Disability, Caregivers, Rehabilitation Services, Collective Health, Chile

## Abstract

In Chile, childhood rehabilitation is recognized as a right supported by regulatory frameworks. However, its implementation presents limitations that restrict access and continuity of care, and the experiences and strategies of women caregivers who use the system have received limited attention in the design and evaluation of public policies. In this context, this article characterizes the therapeutic rehabilitation pathways of children with disabilities in the western area of Santiago, Chile, from the perspective of their primary caregivers. Between August 2023 and March 2024, drawing on a qualitative methodology with an ethnographic approach, participant-observation accompaniment, recorded in field notes, and semistructured interviews were conducted with 13 women. The findings reveal fragmented trajectories marked by multiple barriers to access, a high burden of family management, and discontinuities in care. Four key moments were identified: suspicion and diagnosis, institutional transitions, development of interventions, and therapeutic discharge. Caregivers sustain care processes through everyday strategies that integrate public, community-based, and private forms of support. Within this framework, the article discusses the limitations of current public policies and reinterprets rehabilitation as a contextualized practice sustained by affective and unequal care networks.

## Introduction

Childhood rehabilitation is defined as a planned, intersectoral, and sustained process aimed at optimizing the functioning, development, and well-being of children with health conditions who present or are at risk of experiencing disability.^1^^,^^2^^)^ In line with the International Classification of Functioning, Disability and Health for Children and Youth (ICF-CY),^3^ the World Health Organization recognizes rehabilitation as an essential component of strategies designed to guarantee the right to health for children with disabilities. From an equity and inclusion perspective, these strategies seek to reduce barriers, promote accessible environments, and foster active participation.^4^^,^^5^ This requires solid regulatory frameworks, adequate financing and comprehensive, available, community-relevant services that ensure equitable and high-quality access.^5^

However, in Latin America and the Caribbean, the effective implementation of childhood rehabilitation remains limited due to persistent structural gaps, social inequalities, and institutional fragmentation.^6^^,^^7^ Studies conducted in various countries of the region - primarily Brazil - have documented numerous barriers that affect access to and the quality of these services: incorrect and/or delayed diagnoses,^8^^,^^9^^,^^10^^,^^11^ long waiting lists,^12^^,^^13^ discontinuities in care,^8^ weak intersectoral coordination,^8^^,^^13^^,^^14^ limited recognition of the caregivers role and neglect of their specific needs,^8^^,^^9^ shortage of human resources and material constraints,^13^ among others. The burden of sustaining rehabilitation processes often falls disproportionately on families, especially women, who must undertake an active and often solitary search for fragmented, emotionally demanding, and poorly coordinated services. Along this path, they must navigate public, private, and community resources while sustaining care practices that exceed medical prescriptions, within a setting shaped by class, gender, ethnic, and territorial inequalities.^13^

In Chile, this panorama acquires particular characteristics that mirror many of the trends described above. According to the Third National Disability Survey (ENDISC III),^15^ 14.7% of children and adolescents present some form of disability, with higher prevalence among low-income sectors. Nevertheless, only 14.9% of this population accesses rehabilitation services, despite the existence of a legal framework guaranteeing such access (Law 20.422),^16^ revealing a significant gap between formal rights and the actual conditions for exercising them. Although programs such as the Fund for Child Development Support Interventions (FIADI), implemented within the Chile Crece Más subsystem, and some local initiatives exist, much of the rehabilitation process remains centralized in specialized hospital settings or delegated to private institutions with public funding, including Fundación Teletón, a paradigmatic case of the assistentialist model in the country and the region.^17^^,^^18^ This configuration exposes the system’s structural limitations, which compromise continuity of care and deepen health inequalities in the context of prolonged care trajectories. Moreover, the trajectories of families using rehabilitation services have been scarcely considered in the design and evaluation of public policies, particularly those trajectories that encompass the knowledge, strategies, and tensions caregivers face in their interactions with the health system. Examining these experiences is essential for understanding the dynamics that generate structural barriers and for informing policies that guarantee access to and continuity of rehabilitation from the families’ own perspectives.

Understanding how these trajectories are configured requires an approach that goes beyond institutional description. In this regard, the notion of therapeutic itineraries is particularly useful for analyzing how such pathways unfold in contexts of inequality and fragmentation, such as Chile. Far from constituting linear routes, itineraries are conceived as complex courses, traversed by interruptions, affects, learning processes, and decisions.^19^^,^^20^ From this perspective, the approach to health-illness-care processes (HICP),^21^ which includes rehabilitation,^22^ develops in scenarios where diverse models of care coexist and intertwine in concrete care practices.^23^ Therefore, it is crucial to explore the range of spaces in which the daily lives of children with disabilities and their environments unfold, in order to understand how rehabilitation is produced and what meanings it acquires.

As pointed out by recent studies in collective health, such as those of Venturiello^24^^,^^25^^,^^26^ and Brage,^27^ this analysis requires attention not only to the formal mechanisms available, but also to institutional navigation strategies, everyday resistance, affective learning processes, and the central role assumed by caregivers (mothers, grandmothers, or other women in the family environment). These caregivers act as agents who mediate between different forms of knowledge, manage resources, interpret clinical guidance, and sustain medical-therapeutic relationships under conditions of institutional fragility.

Based on these considerations, the present study aims to characterize the *t*herapeutic itineraries of children with disabilities in the western zone of Santiago de Chile, drawing on the narratives of their primary caregivers. Emphasis is placed on the meanings attributed to the therapeutic process, the strategies deployed to sustain care beyond formal rehabilitation services, and the tensions that emerge in relationships with institutional actors. By rendering these experiences visible, this study seeks to contribute to a critical understanding of child rehabilitation under conditions of structural inequality and to inform the design of public policies that recognize care as a collective practice essential to guaranteeing the right to health.

### Therapeutic pathways in the Rehabilitation of Children with Disabilities: Contributions from Collective Health and Critical Disability Studies

This study is grounded in a perspective that brings together contributions from collective health and critical disability studies to understand health care processes as complex phenomena shaped by power relations, material conditions, institutional frameworks, and diverse forms of knowledge. The notion of HICP^21^ provides a starting point for understanding rehabilitation not as an isolated set of biomedical interventions, but as part of an interwoven network of practices, meanings, and relationships surrounding child health and disability that take shape within the sociocultural contexts of everyday life. In these settings, multiple therapeutic models coexist and interact, shaping singular care strategies from the perspective of medical pluralism.^23^^,^^28^

Within this framework, the concept of itineraries pathway serves as an analytical tool for examining health care processes from the perspective of those who traverse them. According to Alves and Souza,^29^ from a comprehensive perspective, the therapeutic itineraries is understood as a relational and contextual construction composed of the decisions, strategies, meanings, and practices mobilized in the search for care in response to a health need. It is a dialogical and intersubjective process involving a range of social actors, forms of knowledge, affective bonds, and care models, all situated within specific sociohistorical contexts that both constrain and enable HICP trajectories.^20^^,^^30^ In contexts where fragmented and unequal care provision is anticipated, the category of therapeutic itineraries helps explore how caregivers of children with disabilities, using available resources and support networks, construct and negotiate meanings, translate clinical indications, and articulate family, community, and therapeutic knowledge to sustain the rehabilitation process.

From a relational perspective, the care processes surrounding children with disabilities cannot be separated from the material, symbolic, and affective conditions shaping the lives of those who care for them.^31^^,^^32^ Far from being a naturalized role, caregiving is configured as a field of political and epistemic struggle in which frameworks of recognition, resource allocation, knowledge legitimacy, and social visibility of care work are contested. In Latin America, several studies have shown how women - mothers, grandmothers, and other family figures - not only accompany therapeutic processes but also act as active mediators between institutional devices and the needs of the people they care for.^10^^,^^26^^,^^33^^)^ This perspective makes it possible to understand care as an interdependent practice embedded in social and family networks that co-organize daily life around disability, sustained under structurally unequal conditions.^13^

These discussions become especially relevant when considering child rehabilitation as a field shaped by normalizing discourses and expectations of functional adherence. Critical disability and childhood studies^34^^,^^35^^,^^36^^,^^37^ have noted how traditional rehabilitation models tend to organize the lives of children with disabilities around prescribed developmental trajectories, demanding the correction of deviations from an ideal of functionality. Such expectations operate not only on children’s bodies but also on families, who must often adapt to rigid institutional frameworks that transform domestic environments into spaces of constant professional intervention. In this sense, the position of the family - particularly mothers - within the therapeutic chain of responsibilities is problematized, along with the effects these demands produce in their life trajectories.^33^^,^^38^^,^^39^^,^^40^^,^^41^

Latin American perspectives highlight that disability is not exclusively an individual condition but a relational experience shaped through interactions among people, institutions, and territories.^34^ Thus, examining rehabilitation through the lens of therapeutic itineraries means recognizing that this process unfolds within a field of disputes-between forms of knowledge, models, and actors-where what is considered “therapeutic” is redefined beyond the professional consultation. As studies in the region have shown,^11^^,^^13^^,^^24^^,^^25^^,^^26^^,^^27^ these trajectories are deeply shaped by relationships of gender, class, territory, and affect, forming a practical pedagogy of care that functions simultaneously as a mode of resistance and a means of producing knowledge from lived experience in contexts of structural inequality.

## Methods

This study adopts a qualitative approach aimed at understanding therapeutic itineraries of child rehabilitation from the perspective of those who sustain daily care processes. It is grounded in the interpretive paradigm,^42^ which recognizes these trajectories as intersubjective constructions situated in contexts shaped by power relations, cultural meanings, and institutional structures. Guided by an ethnographic approach,^43^ semi-structured interviews, participant observations, and field notes were conducted, enabling the capture of caregivers’ narratives as well as their interactions, silences, and practices. The sample was constructed through purposive sampling and snowball techniques, including 13 primary caregivers (12 mothers and one grandmother) responsible for children aged 1 to 9 with permanent disabilities, residing in the western area of Santiago and users of the public health system.

Fieldwork took place between August 2023 and March 2024 and included home visits, accompaniment to rehabilitation centers, and situated observations in community settings. A total of 30 interviews were conducted, each lasting between 60 and 90 minutes, recorded and fully transcribed. The interview guide was flexible and aimed at narrative reconstruction regarding access, institutional relationships, and meanings of therapeutic processes. Interviews were conducted primarily in participants’ homes or everyday spaces, allowing narratives to be anchored within the contexts where care practices and family dynamics occur. These settings fostered an atmosphere of trust and the production of detailed narratives; however, in some cases, they posed limitations related to varying conditions and timing or the presence of family members, factors considered in the analysis and interpretation of data. Thematic analysis was conducted using open, axial, and selective coding with ATLAS.ti, in dialogue with principles of grounded theory.^44^ Graphical diagrams and narrative reconstructions of itineraries were developed and validated with participants, strengthening interpretive coherence.

Reflexivity involved acknowledging the researcher’s position as a speech therapist working in the public system and as a man in a feminized field. This positionality required sustained critical engagement through collective discussions, analysis of field notes, and validation of the findings.

The study received approval from the Research Ethics Committee for Human Subjects of the Faculty of Medicine, University of Chile (Project No. 027-2023). All participants were informed of the study’s objectives, scope, and procedures, including recording, transcription, and academic use of the narratives. Confidentiality was ensured through the assignment of alphanumeric codes (e.g., C1), unlinked from personal data, and by removing identifying information. Records were stored in protected devices with restricted access, ensuring anonymization throughout the coding and results presentation process. These measures safeguarded participant privacy and the analytical coherence of the material used.

The results presented here form part of the doctoral work of the principal author in fulfillment of the requirements for the Doctor of Public Health degree at the University of Chile. The broader dissertation is titled *“*Rehabilitation Processes of Children with Disabilities in the Western Zone of Santiago de Chile: An Approach to the Experiences of Their Primary Caregivers from Therapeutic Itineraries.”

## Results

Based on the analysis of primary caregivers’ narratives, four key moments in the therapeutic itinerariess of children rehabilitation were identified: 1) suspicion and confirmation that something is happening**:** the experiences that initiate the pathway; 2) the pilgrimage to reach rehabilitation: institutional transitions, waiting times, and self-management; 3) the development of therapeutic interventions: meanings, tensions, and re-signification of rehabilitation; 4) continuity and open-ended projections of the rehabilitation process: the tension between intermittent support and therapeutic discharge. These moments should not be understood as rigid chronological stages but as analytical categories that help characterize the complexities of the rehabilitation pathways, acknowledging their diversity and the specificities of each case.

### Suspicion and confirmation that something Is happening: experiences that Initiate the pathway

The rehabilitation pathways analyzed in this study usually begin during the early years of life. In some cases, they are triggered by diagnoses made during the pre- or perinatal stages, often associated with genetic conditions or prematurity. In others, suspicion emerges in everyday life through a recurring perception by caregivers or close relatives that “something is not right.” Although these experiences differ in their origin, both mark a turning point in the family’s trajectory, initiating a process of searching, uncertainty, and reorganization of daily life.

Early diagnoses, far from being experienced as merely clinical acts, take shape as what Osorio Carranza refers to as biographical disruptions:^45^ moments that profoundly alter expectations, temporalities, and family roles, while also redefining the status of the child with disability for both the health system and the family. For caregivers, learning the diagnosis often entails a double or even triple concern-especially during prolonged postpartum hospitalizations-since they must manage the care of other young family members and assess the resources available for medical supplies in contexts marked by unemployment or informal work.

Within this context, caregivers report experiences characterized by tensions with healthcare teams, frequently expressed through forms of obstetric violence,^46^ distrust, or recommendations that dismiss their convictions and emotions. The medical figure often appears as a bearer of technocratic knowledge that, at times, imposes decisions without considering the circumstances caregivers are undergoing.

“*They told me the law protected me and that I could have an abortion, because my baby wasn’t going to be born alive. With that syndrome [trisomy 18], babies didn’t survive. I told them no - that if my baby was going to die, I would accept it, but I wasn’t willing to be the one to end her life*.” (C5)

“*The midwife told me that my pregnancy was extremely high-risk, that it was almost impossible for it to reach full term, and that she wasn’t even going to issue me a prenatal card. I asked her for an order for an ultrasound, and she said: ‘Sure, but don’t get your hopes up - it might not even be there.’*” (C6)

“*They asked me if I wanted her or not, just like that: ‘Are you going to keep the baby girl, or are you giving her up for adoption?’ I told them they were crazy, that she was my daughter and I wasn’t going to give her up*.” (C8)

In this stage, the coexistence of care and institutional neglect processes becomes evident ,^23^^,^^47^ where indifference or dismissal of some professionals can be considered as manifestations of structural neglect that reproduce hierarchies of class, gender, and knowledge.^47^ In response to these situations, women deploy strategies of resistance, such as rejecting medical recommendations, seeking second opinions, or activating family networks to cover the costs of private examinations when economic resources and their type of health insurance allow it. These actions not only express agency but also make it possible to uphold affective and ethical decisions under conditions of precarity.

In other cases, the pathway does not begin with a medical diagnosis but with a sustained suspicion that arises in domestic life. These signs often appear between 6 and 24 months, when caregivers-through observation, comparison, or intuition-perceive that their child’s development does not align with normative expectations.

“*Everything was normal, he drank his milk, he was a healthy baby to me until he was about five or six months old, when I’d leave him there on the bed and he’d just stay still. If you put him on his side, he would flop to the sides, and that’s when we realized he couldn’t hold his little head up*.” (C1)

These signs are not always received with certainty. Often, caregivers move between denial, doubt, self-deception, or external pressure. Family networks may provide emotional support, but they can also impose mandates that hold caregivers responsible for the diagnosis or for “not having noticed it earlier.” As analyzed by Runswick-Cole et al.,^39^^,^^40^ an imperative of vigilance and management of child development is placed upon them, one that shapes their social valuation as mothers.

*“I didn’t really pay attention to it because he was just a baby… When he was about a year and five months old, my partner’s aunts kept telling me, ‘That’s not normal.’ But I would say, it is normal, because they told me he was born healthy.”* (C4)

The health system, instead of acknowledging these suspicions, often delegitimizes them. As reported by Puga et al.,^11^ experiences of institutional minimization delay timely diagnoses and limit access to early interventions. This not only amplifies uncertainty but also deepens the institutional neglect processes described by Hersh-Martínez.^47^

*“The doctor at* [name of hospital] *told me it was all pure morning, that he was a completely healthy baby.”* (C3)

*“At the hospital I had bad luck the first few times. The first time they just looked at her like that and said, ‘That’s just grandma stuff.’ The second neurologist said, ‘I’m not going to do anything to her so she doesn’t cry,’ and they didn’t do anything.”* (C2)

Faced with the lack of accurate diagnoses, delays in care, depersonalized treatment, and the negotiations between expert knowledge and caregivers’ experiential knowledge, caregivers take on an active role in searching for health services that meet their needs. They seek consultations outside the public system, interpret medical reports, look for guidance within informal networks, and document their children’s behavior to accumulate evidence. These actions, described by Brage as everyday tactics,^27^ reflect a way of sustaining care through lived experience, even without an official diagnosis and amid institutional fragmentation.

*“I wanted to get a second opinion from the neurologist. Every time I had a check-up with her, she gave me a different diagnosis: four or five months would go by and she’d change her mind… We went to a private neurologist because we wanted a referral to see if it was possible for her to get into Teletón. I didn’t want her to lose what she had already learned. I wanted them to keep helping her, or at least tell me how I could keep working with her at home.”*(C13)

In this way, rather than merely resisting, caregivers transform neglect into practical and affective learning that contributes to the development of what Pava-Ripoll terms emotional capital,^33^ understood as a form of affective knowledge that combines reflection, intuition, and action to confront the challenges of the process.

Thus, this initial stage of the itinerary not only inaugurates the relationship with the care system but also constitutes a formative experience that shapes the caregiving role. Ambivalent emotions-such as distress, guilt, or relief-intertwine with practical actions of containment, searching, and advocacy. As Goodley^36^ suggests, in these contexts the diagnosis, or its absence, becomes a milestone that cannot be reduced to a marker of deficit or an expression of vulnerability, as it entails a series of affective reconfigurations, redefinitions of family priorities, disputes over meaning, and efforts that reveal a transformative potential for everyday life and reshape the place of disability within it.

### Pilgrimage to rehabilitation: institutional transitions, waiting times, and self-management

Following the initial suspicion or diagnosis, families enter a stage marked by an intense and fragmented journey through different levels of the health and education systems in search of adequate therapeutic support. Access to rehabilitation is conditioned by structural factors such as health insurance, the territorial organization of services, the availability of personal networks, and the capacity to decode information within an opaque system. Some caregivers manage to enter through referrals from primary care or hospital services, while others rely on informal references, known professionals, or private consultations.

This stage of the pathway can be understood as an “institutional journey,” in which caregivers take on tasks that exceed the passive role of service users, as they coordinate referrals, manage documents and transportation, and provide emotional support to the family group. In line with what Alves^19^ and Menéndez^23^ propose, these modes of navigating the system are not mere movements between services but spaces where distinct rationalities-medical, domestic, and bureaucratic-intersect and give rise to concrete forms of care. Thereby, the pilgrimage not only exposes systemic inefficiencies but also shows how families co-produce care and actively participate in the social production of the rehabilitation process in contexts of fragmentation.

A central finding at this stage is the function of the medical diagnosis as a key to accessing therapies and social services. More than a clinical tool, the diagnosis becomes an institutional credential that legitimizes the demand and determines the types of available support. This phenomenon aligns with the processes of legitimization or validation of demand described by Gerhardt,^20^ Venturiello,^24^ and Brage,^27^ in which the recognition of a need for care depends more on the capacity to meet bureaucratic requirements than on the urgency or complexity of the case. In this way, families are forced to accumulate legitimacy before the system in order to be recognized as bearers of a valid demand, which redefines the very meaning of the care process.

*“She told me, ‘The thing is, we can’t admit him without a diagnosis, a report, an exam, or something like that.’ So I told her okay, and then she said, ‘If you can take him back to a (private) neurologist, that would be good. If not, we’ll put him on the waiting list for the doctor anyway, but that could take a long time. If you have the possibility of taking him somewhere else, do it.***’”** (C11)

At the same time, the public health system - weakened by resource shortages and long waiting lists - implicitly fosters an articulation with private providers, including those in the non-profit private sector (foundations and corporations operating with state funding), as an institutional compensation strategy. As a consequence, the economic and logistical burden on families intensifies, as they must go into debt or activate personal networks to secure timely care, thereby deepening social and territorial inequalities.

*“When the care at the foundation ended, I thought, ‘Now what?’ We had to wait for them to contact us again to know what to do with my son, which therapist to take him to. Then we went to the neurologist and she told me, ‘This isn’t my area anymore; now it’s something genetic.’ But there’s nothing available on the genetics side, so you have to do it privately.”* (C1)

Out-of-pocket expenses emerge as a threshold for access. Families face payments for examinations, transportation, or private therapy sessions. This silent privatization of care occurs in a context of precarity, where public policies do not guarantee effective continuity. This operation mode - often naturalized as a necessary complement - reinforces what Menéndez^23^ and Hersch-Martínez^47^ describe as structural neglect, understood as a form of covert privatization of care that shifts onto households the responsibility for ensuring therapeutic continuity.

*“The ADOS test* [Autism Diagnostic Observation Schedule] *cost between 140, 130, 120 thousand pesos. And she, I think she charged me 100 thousand. If I didn’t get it done, I couldn’t take him to the therapist.”* [$100,000 CLP is approximately $110 USD based on the 2023-2024 average exchange rate]*.* (C7)

*“She told me I’d have to do it on my own. So I went to an institute they told me was the most specialized place. The first test cost 200 thousand pesos, the next one 400 thousand. The last one was around 900 thousand, and it came back normal.***”** (C1)

At this lack of protection, caregivers deploy a variety of strategies: they register in multiple centers, file complaints, rely on personal contacts, seek shortcuts, or reorganize their finances. These actions express a form of agency constructed within neglect that, as Venturiello^26^ argues, constitutes a pedagogy of care-that is, a practical and relational knowledge learned through experience that enables caregivers to sustain support beyond institutional prescriptions. This capacity to act amid uncertainty also involves an affective and ethical dimension that, in dialogue with Pava-Ripoll,^33^ forms part of and contributes to caregivers’ emotional capital by mobilizing energy and commitment to maintain care in the absence of structural support. Nonetheless, these learnings entail physical and moral costs that reveal the limits of such agency.

*“I’m the one who books all the appointments, the one who explains everything whenever they change therapists, the one who fights for the referrals. Sometimes I feel more like a social worker than a mom.”* (C6)

*“I was already so exhausted, frustrated. I even called the Ministry of Health to find out if they could do something, because I was honestly overwhelmed. We checked how much private therapy would cost, and it was something like forty thousand pesos per session, three times a week. It was insanely expensive, but I kept thinking: ‘It doesn’t matter, we’ll figure it out so she gets the therapy she needs. We’ll do whatever we have to do to pay for it.”* (C11)

This trajectory also leaves emotional and physical traces. Caregivers describe symptoms of exhaustion, insomnia, anxiety, and bodily pain. Guilt emerges as a constant: for not having acted earlier, for not being able to handle everything, for feeling that something is always missing. These experiences confirm that care is produced within a terrain of tension. While Thomas^48^ conceives care as an ethical practice aimed at sustaining life and interdependence, the narratives gathered here reveal the limits of that ethic when care is carried out without the material or institutional conditions needed to sustain it. In this sense, Kittay^31^ expands the debate by situating dependency and support for the caregiver as central dimensions of relational justice, arguing that care is not possible if those who care are not, in turn, cared for. The participants’ voices thus reveal a relevant paradox in which the very act that preserves life also generates exhaustion, showing that the ethics of care must be accompanied by policies that acknowledge and sustain its material and collective dimensions.

“*There were weeks when I didn’t sleep at all. So the neurologist told me I needed to get some rest.”* (C6)

*“I’m tired, but if I don’t do it, no one will.”* (Fieldnote, accompaniment with C2)

This stage cannot be reduced to a mere preliminary procedure. It is a complex experience in which institutional strain intersects with forced learning, ethical decisions, and networks of support. Throughout this journey, caregivers not only confront material barriers but also reformulate their relationship with the healthcare system and reconfigure the ways they inhabit care-shaped by social mandates, accumulated inequalities, and affective availability which, while sustaining rehabilitation, also exposes its limits.

### The development of therapeutic Interventions: meanings, tensions, and the re-signification of rehabilitation

After the initial stage of institutional mobilization, caregivers enter a new terrain: therapeutic interventions. Far from representing a moment of containment, this phase is experienced as an extension of previous demands. The institutions involved include a variety of centers, both public and private: Family Health Centers (CESFAM), municipal programs, hospitals, national institutes, private foundations, independently practicing professionals, among others. In some cases, treatments take place in the home through domiciliary modalities. Rather than being guaranteed, therapy sessions depend on multiple factors: professionals’ availability, diagnostic criteria, funding agreements, and, above all, the daily management carried out by families. Added to this, the limited time allotted for appointments and the frequent turnover of professionals, which weakens therapeutic continuity.

*“It didn’t use to be like that. The previous physical therapist gave me all the appointments for the month and saw us continuously, for almost a year. But the new professional put us on a plan of only eight sessions. If there’s no progress, they drop you***.”** (C5)

Added to these limitations are other barriers that hinder the effective exercise of the right to rehabilitation. Difficulties in accessing sessions include excessive distances between the home and service centers, lack of accessible transportation, inability to reorganize schedules due to informal employment, absence of companion, or conflicts with other scheduled school or medical activities.

*“…and sometimes we ended up missing the appointment, because sometimes it takes me three hours to get to the institute. In the metro, the hardest part is the elevators - they’re often out of order, and I have to go all the way to the next station.”* (fieldnotes, accompaniment with C9)

*“He still needs more support, and one speech therapy session every two weeks isn’t enough. They’ve told me that if I can look for more help, I should. But so far, I don’t have the time or the money to pay for more therapies, or to have someone come to the house. If I take him myself, I lose the whole afternoon of work. And on top of that, I stop working and still have to take care of my other daughter.”* (C4)

These difficulties are compounded by the need to co-finance treatments. Private therapies, transportation, food during travel, and materials for home-based exercises represent significant out-of-pocket costs. Although families should have guaranteed care through the public system, they must assume ongoing expenses to ensure continuity and quality in the rehabilitation process.

*“Back then we weren’t receiving any disability pension, so we tried to get FONASA to provide more coverage - not money, but more sessions and hours. We paid for a lot of physical therapy sessions and, since we were going to a medical center, her dad would buy the vouchers under his name so my daughter could be seen. We also bought vouchers under her sister’s name, my name, my mom’s… anything so she wouldn’t miss her therapies.”* (C6)

*“Between transportation, food, and the sessions, my whole week’s money disappears, so I organize different things to raise funds. My husband and I do raffles, sell ‘completos,’ anything to bring in some income.”* (fieldnotes, accompaniment with C5)

Experiences of care within pediatric rehabilitation are affected by programs with limited coverage depending on age or available resources, which lead to frequent turnover of professionals and changes in therapeutic approaches. In this context, caregivers develop diverse assessments: in some cases, they highlight the building of relationships grounded in empathy, active listening, and recognition of their role, which helps create spaces of trust and emotional support amid instability. In others, they encounter tense relationships with technical teams, marked by rigidity, impersonal treatment, or the devaluation of their experience, reinforcing a sense of exclusion and discontinuity in the care process. As Venturiello^25^^,^^26^ and Gerhardt^20^ argue, the therapeutic itineraries is not sustained solely by technical prescriptions but by everyday negotiations in which the recognition of the caregiving role is contested. In response to these tensions, caregivers deploy strategies to assert their voice: they observe, compare, adapt recommendations, and, when they deem it necessary, interrupt or modify the treatment.

*“I told the therapist that she had already gone through that stage, but they treated her as if she were just starting. She would get bored; she did the exercises quickly just to get it over with. Sometimes it’s hard to get professionals to listen, as if we didn’t know what we’ve lived through with our kids.”* (C2)

Rehabilitation is not limited to what is prescribed by services: it is lived and adjusted according to everyday life, to what is perceived as useful, feasible, or sustainable. In this context, families extend it into the home, the school, or the community, incorporating daily activities, play, sensory stimulation, or adapted exercises. They also turn to complementary therapies such as hippotherapy or regular use of swimming pools, most of which are self-managed or facilitated through local networks. These practices, in dialogue with medical pluralism,^21^^,^^28^ show how families combine biomedical, familial, and community-based forms of knowledge to configure new modes of care. In doing so, caregivers articulate experiences and knowledge, transforming rehabilitation into a distributed practice aimed at sustaining care within the limits possible.

*“When she was about a year and two months old, we started hippotherapy. It’s like with the therapist: we go through these cycles where they discharge us, then half a semester later they call us back, and that’s how we keep going. She loves it, and it really helps her.”* (C6)

*“A friend invited me to a workshop in a heated pool, where on Saturdays they open up three lanes for children with disabilities… From the very first day, he was so happy in the water. He would let go on his own, float with the pool noodles, and everyone laughed. It did him a lot of good, especially for his muscles. The sessions were two hours long.”* (C1)

Within this network of efforts, educational institutions also become spaces where the therapeutic dimension is partially sustained. Some families find in the school - through the support of health professionals or special education teachers - a continuity that they often cannot obtain from health centers. However, although these arrangements are valued by caregivers, their design and conditions are not intended to replace rehabilitation processes, which strains the expectations placed on schools and reflects the fragility of the institutional framework of available services.

*“So far, she only has a speech therapist at school. She hasn’t sent the notebook home, so now she’s going to have to start sending it with the activities for stimulation.”* (C1)

*“At school, the teacher* [therapist] *told me he’s made a lot of progress. When he first started, he already knew the vowels. One day she wrote them on the board and he said them all: a-e-i-o-u. I got emotional because I had spent months practicing them with him at home. For me, that’s a huge achievement.”* (C3)

The development of therapeutic interventions takes shape as an extended and non-linear process that exceeds institutional boundaries. For these families, rehabilitation does not mean only attending formal sessions; rather, it involves articulating multiple actions distributed across the home, the school, the community, and health centers. Within this network, families combine supports, strategies, and resources, integrating everyday life into therapeutic practice and adapting it to conditions of possibility and sustainability in contexts marked by inequality. This movement transforms the very meaning of disability, as it is no longer oriented only toward correcting or compensating for a deficit but toward reorganizing life around other forms of value, relationship, and agency. In this sense, Goodley^36^ and Runswick-Cole^39^ argue that disability can be understood as a space of social production of affects and relationships, where dependency is reinterpreted as interdependence and the possible forms of autonomy are expanded. From this perspective, caregivers’ practices reveal an affirmative potential: as they sustain rehabilitation, they also reinvent ways of living and reconstruct the notion of development outside the normative ideal.

### Continuity and open projections of the rehabilitation process: the tension between intermittent support and therapeutic discharge

Most of the trajectories analyzed in this study involve long-term rehabilitation processes, often extending beyond four years. Caregivers describe progress, setbacks, and periods of stagnation, in a pathway that does not follow a linear pattern. The process is interrupted and resumed, relocated, or adapted depending on each child’s development, clinical decisions, and the families’ own perceptions. Throughout the interviews, different forms of closure or suspension were identified. Some correspond to a formal discharge, issued when a stage is considered complete or the need for care resolved. Others appear as temporary interruptions related to professional turnover, lack of available appointments, or institutional saturation. In several cases, caregivers interpret discharge not as the result of a comprehensive evaluation but as an administrative decision. Voluntary interruptions by families were also described, motivated by the perception of limited progress or disagreement with the therapeutic approach.

In response to this situation, caregivers develop strategies to sustain continuity despite the formal closure of the process. They seek new referrals, pay for private sessions, contact familiar therapists, or strengthen support within school and community settings. Therapeutic work, far from ending, is displaced, shifted to new spaces, transformed in its modalities, and embedded within other relationships.

*“Now that the therapist discharged him, I’m looking for a foundation that’s free, because I can’t afford those kinds of weekly expenses. It’s a lot of money, considering it’s several therapies. For example, if he needs a speech therapist, an occupational therapist, it’s about 50,000 pesos a week, and I just don’t have the resources.”* (C7)

This persistence can be read, following Menéndez’s^23^ formulation, as a form of expanded self-care, understood as a collective process that reconfigures the boundaries of the system and enables the continuity of care beyond its formal coverage. Within these dynamics, the caregivers’ everyday work sustains the continuity that institutions interrupt, shaping a parallel network in which rehabilitation is co-produced both within and beyond the system.

In this phase, expectations about the future and the meaning of the process acquire a central place. Over time, the goal shifts from normalization toward situated forms of accompaniment and attainable functional objectives, where each everyday achievement holds its own value. This transformation also entails a new way of understanding disability - one that moves away from deficit-centered frameworks and toward the particular rhythms of each child. It implies a relational and affective understanding of disability, akin to Goodley’s^37^ notion of *dis/ability*, in which difference is taken as a part of life rather than a deficiency to be overcome. However, this shift is neither absolute nor uniform. Acceptance coexists with ambivalences, frustrations, and the difficulties of acknowledging that certain conditions will not disappear.

*“With these kids, everything is step by step. It’s not about getting your hopes too high, but about being there with them as they make their own progress. As moms we want the best, but it’s better to appreciate every little milestone than to get frustrated waiting for something more. In my case, I look at my son and to me he’s healthy: he doesn’t need medication, he eats well, he’s active. That’s what it is - celebrating his achievements with him and looking for support wherever we can find it.”* (C1)

*“Even today, I still struggle. It’s really hard for me to accept that she’s not sick, that this is a condition, that she’s going to keep being this way. That’s what’s hardest for me.”* (C2)

In this way, although clinical devices may establish a formal endpoint, the therapeutic itinerariess that accompany the development of children with disabilities continue beyond institutional boundaries. Families, drawing on the resources available to them, mediate between the clinical ideal of discharge and the ongoing continuity of care. In the terms proposed by Osorio Carranza,^45^ rehabilitation becomes integrated into the family’s biographical trajectory and extends as a vital process rather than a purely health-related episode, adapting to what is possible and meaningful within each experience.

## Discussion

The therapeutic itinerariess of childhood rehabilitation in the western area of Santiago, as they emerge in caregivers’ narratives, show that rehabilitation cannot be understood as a linear or exclusively clinical trajectory. Rather, it is configured as a socially, politically, and affectively dense practice, shaped by interruptions, moral decisions, and disputes over meaning. In dialogue with Latin American contributions on therapeutic itineraries, the findings confirm that families do not merely move through services; instead, they co-produce rehabilitation through affective, ethical, and organizational strategies that compensate for institutional neglect.^13^^,^^20^^,^^21^^,^^23^^,^^24^^,^^27^ From this perspective, rehabilitation is understood as a relational process that combines professional and family knowledge, sustained by the learning, bonds, and resources that families mobilize in unequal contexts.

In the case of Chile, although the country adheres to international disability rights frameworks and has a normative structure for rehabilitation,^16^ the exercise of this right remains conditioned by bureaucratic requirements, dependence on diagnosis, type of insurance coverage, territorial location, and families’ economic capacity. This produces access circuits shaped more by families’ ability to manage referrals, activate networks, and finance services than by universal guarantees, reproducing the structural neglect described by Menéndez^23^ and Hersch-Martínez.^47^ This pattern aligns with findings from other Latin American contexts, where diagnostic delays, discontinuities, and weak intersectoral coordination persist.^9^^,^^10^^,^^11^ However, while countries such as Brazil have achieved some articulation across levels of care through the Unified Health System, in Chile the subsidiary logic of the system results in a “third-sectorization of the public sphere,” delegating therapeutic continuity to foundations and private providers. Unlike the scenarios described by Pedrosa et al.,^8^ where public networks seek to articulate community programs, the Chilean experience unfolds in a mixed and fragmented system in which rehabilitation largely depends on domestic labor and families’ ability to pay. This produces a form of concealed privatization of care that reinforces class and gender inequalities, lacking the institutional counterweights observed in other countries of the region.^10^^.^^27^

In this scenario, caregivers assume a decisive role in sustaining the process. During their search for care, their pathways resemble an institutional pilgrimage in which they coordinate referrals, manage documents, and cover treatment costs. This labor, invisible within health policy, constitutes a form of political agency that transforms neglect into action. In dialogue with Venturiello’s study on therapeutic itineraries,^24^ women develop what can be understood as pedagogies of care-practical and relational forms of knowledge learned through experience that allow them to sustain processes beyond state capacities.

The analysis of therapeutic interventions shows that caregivers broaden the notion of rehabilitation by incorporating domestic, community, and school-based practices - such as play activities, exercises, alternative therapies, and educational support - that configure a distributed and plural process extending beyond biomedical prescription.^25^^)^ This finding aligns with the medical pluralism described by Menéndez^21^^)^ and taken up by Venturiello,^25^ in which biomedical, family, and community rationalities coexist and are reconfigured according to local conditions. Within this context, the community sphere, although less visible than institutional structures, consolidates itself as a space in which care, support, and accompaniment are sustained. Extracurricular workshops, informal networks, neighborhood centers, and practices of cooperation among women form parallel care infrastructures.

However, unlike Brazilian experiences that have integrated community therapies into public programs,^8^^)^ in the Chilean context existing family and community networks receive minimal institutional recognition, and continuity is built amid uncertainty. Families are not isolated, yet their collective action lacks public validation. Rather than secondary supports, these initiatives express a collective dimension of care that sustains processes at the margins of the formal system. These contrasts illustrate the diverse expressions of care in Latin American contexts of childhood disability, where collective and relational forms of care coexist with more individualized and privatized experiences. In both scenarios, the feminization of care labor and structural inequalities remain central axes shaping access, continuity, and the meaning of therapeutic processes. From the perspective of collective health, acknowledging their potential implies recognizing these actors as legitimate contributors to the social production of health.

In line with this reading, Meyer et al.^1^ and Negrini et al.^2^ propose understanding rehabilitation as an intersectoral, sustained, and wellbeing-oriented process, rather than a collection of clinical interventions. Although international and national frameworks recognize rehabilitation as a right and an integral strategy, Chilean trajectories reveal an institutional configuration that does not coordinate health, education, and social care but instead fragments responsibilities and limits coordination. From this perspective, the gap between normative ideals and practical implementation becomes visible, exposing the distance between the formal right and its effective exercise in unequal settings. The Chilean experience, therefore, shows an institutional landscape that fragments responsibilities and lacks coordination across health, education, and social care. This mismatch between normative definitions and practical execution underscores the distance between formal rights and their effective realization in settings marked by inequality.

Toward the end of the pathway, many caregivers shift their expectations from recovery toward an understanding of rehabilitation as lifelong accompaniment, beyond normalization.^34^^,^^49^ This shift resonates with Osorio Carranza’s^45^ conceptualization of the biographical trajectories of care and with approaches that conceive disability as a relational and affective experience.^36^ From this perspective, families’ practices challenge the notion of “normal” development that underpins much therapeutic and educational intervention. As Remorini and Rowensztein^49^ argue, ideas of normality in child development are not biological facts but historical constructions that guide classification, measurement, and hierarchization of bodies and learning. The findings of this study show how caregivers resist this normative frame, valuing everyday progress according to each child’s rhythms and possibilities rather than clinical parameters of progress. In this sense, the reconfiguration of rehabilitation in Chilean trajectories is not merely an adaptation to resource scarcity but an ethical and epistemological redefinition of care that questions knowledge hierarchies and normalization expectations.

Although this study did not aim to produce a systematic comparative analysis by types of services, relevant differences were observed across itineraries depending on program characteristics, access to extended family networks, and the educational modalities in which children participated (special schools, school integration programs, or others). Caregivers with stronger community ties or involvement in organized disability spaces (such as foundations or neighborhood centers) demonstrated greater capacity for management, mobility, and emotional support, confirming the relevance of social networks as determinants of the therapeutic itineraries.^26^ These experiences underscore the need to strengthen community-based rehabilitation spaces within public policy as legitimate components of health networks, recognizing their role in the collective production of wellbeing. This represents an analytical direction to be further explored in future studies, particularly regarding how these variables mediate the actual possibilities of therapeutic continuity.

From a public policy perspective, these findings question the real scope of programs such as Chile Crece Más or local rehabilitation networks, whose implementation is hindered by the lack of intersectoral coordination and the weight of administrative criteria. Diagnosis becomes a key to access rather than part of ongoing support, and coverage remains determined by insurance status and families’ ability to pay. This situation, also observed by Puga et al.^11^ in other Latin American countries, acquires particular intensity in Chile due to the weakness of the public system and the concentration of services in privately run institutions funded by the state. Primary care, with limited capacity to address childhood disability, fails to articulate continuous care, while specialized services are concentrated in high-complexity centers with restricted coverage and uneven discharge criteria. From the perspective of collective health, this demonstrates that the formal universality of the right to rehabilitation does not guarantee health justice unless the relational and community dimensions of care in contexts of structural inequality are recognized.

The qualitative nature and territorial focus of this study limit generalizability, though they allow the exploration of affective, moral, and political dimensions that are difficult to capture through other approaches. Accompanied observations added depth to the understanding of pathways, yet expanding their scope would be necessary to observe institutional interactions in real time. Future studies could also compare trajectories across territories or service modalities to examine how socioeconomic and network factors shape the continuity of care.

In conclusion, the pathways analyzed show that the continuity of rehabilitation does not depend on the health system but on caregivers’ daily, affective, and ethical labor. In contexts of institutional fragmentation and inequality, families co-produce rehabilitation through management, caregiving, and learning strategies that sustain processes where the state is absent. This experience reveals that rehabilitation is not a technical practice but a social and political action that redefines the meanings of disability, care, and citizenship. In light of this, it is necessary to advance toward public policies that recognize care as a constitutive dimension of the right to health and strengthen community networks as legitimate spaces for the production of wellbeing. Guaranteeing rehabilitation also requires guaranteeing the social conditions that make care possible.
